# Supplementary figures and images for: Estimation of regional polygenicity from GWAS provides insights into the genetic architecture of complex traits
Source: PLoS Comput Biol. 2021 Oct 21;17(10):e1009483. doi: 10.1371/journal.pcbi.1009483 (PMC8562817; doi:10.1371/journal.pcbi.1009483)

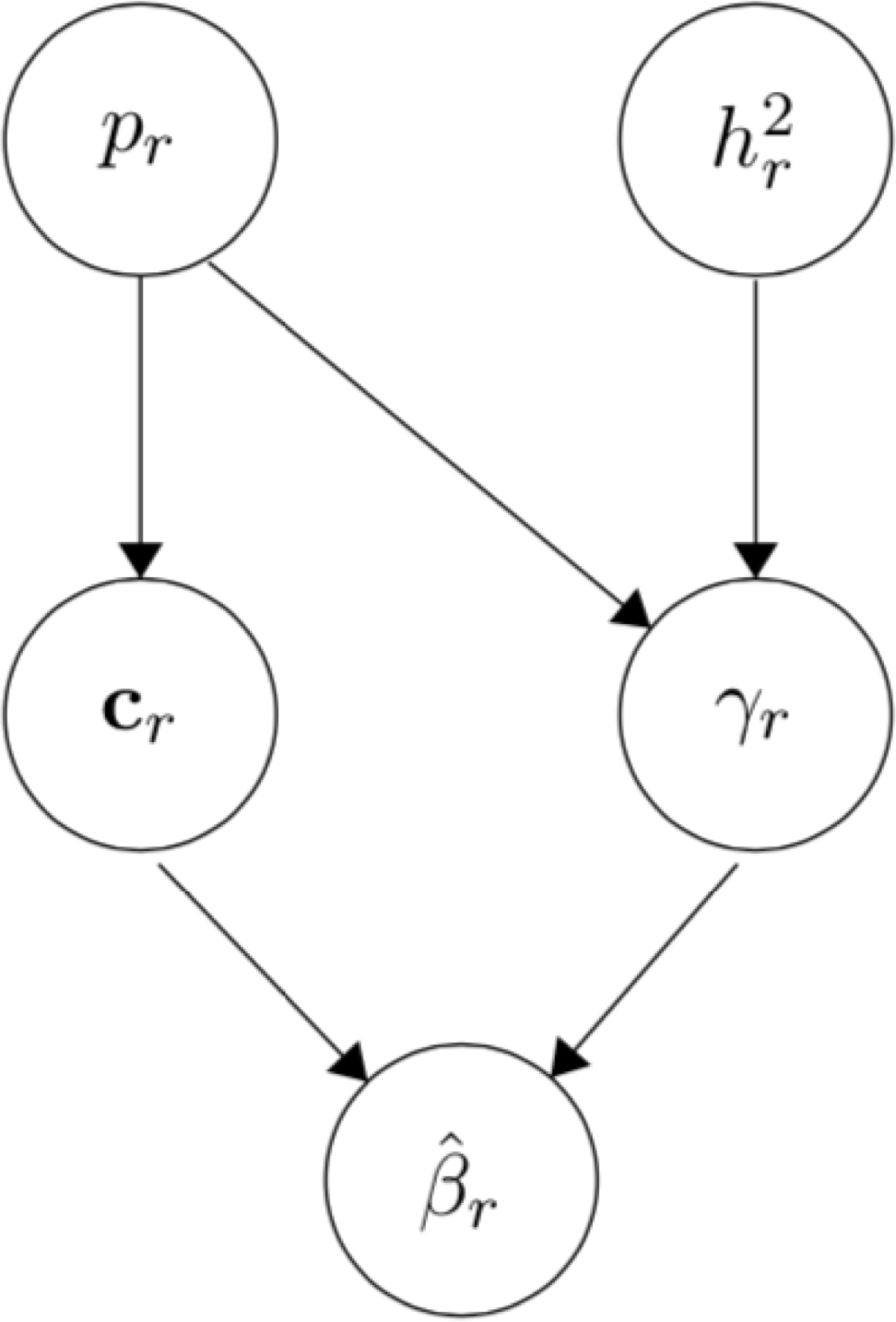

Supplement: S1 Fig — Directed graphical model diagram for BEAVR. (TIF) [file pcbi.1009483.s001.tif]

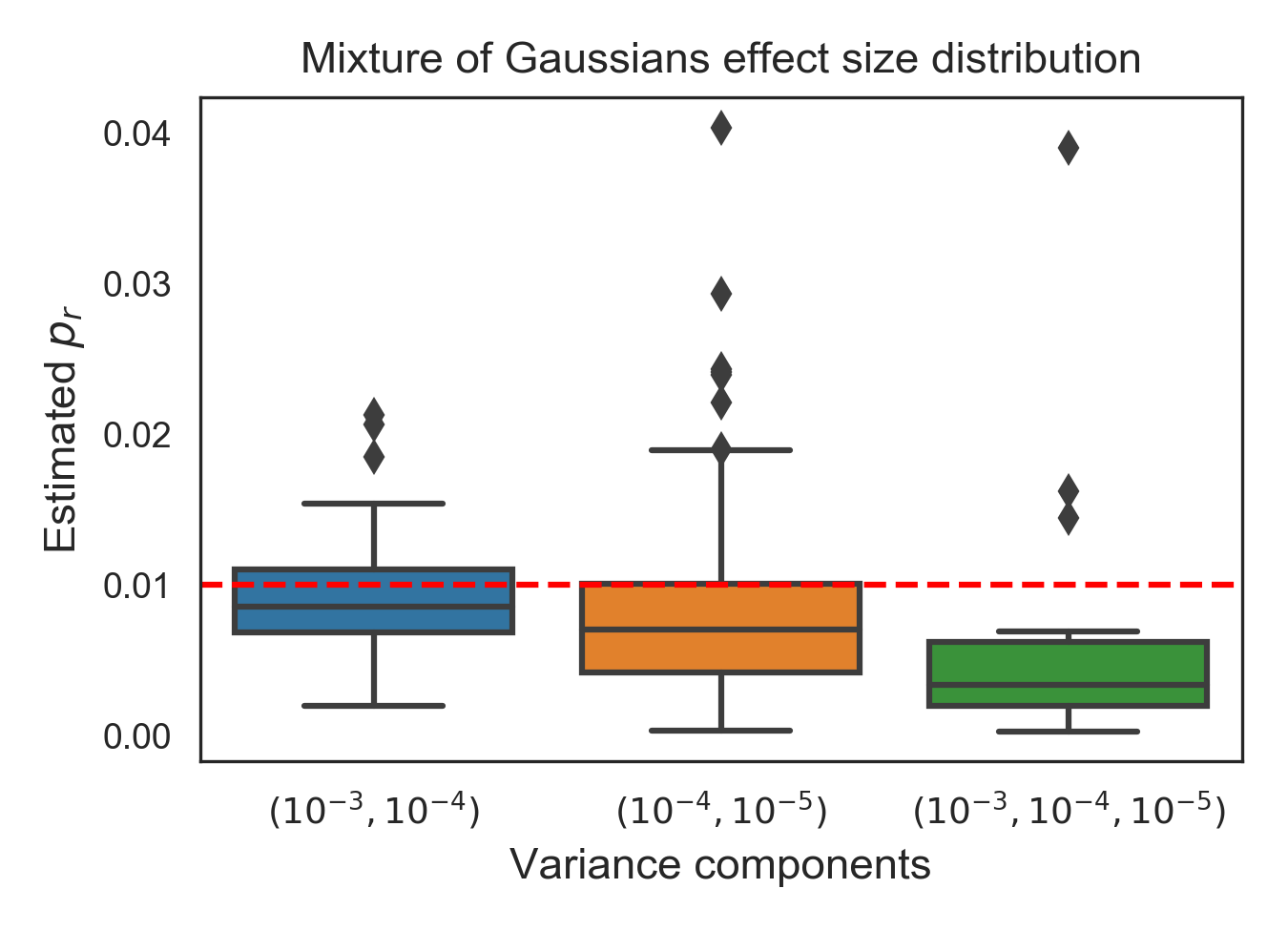

Supplement: S2 Fig — We simulate effect sizes from mixture of Gaussian distributions with the following set of variance components: [1 × 10^−3^, 1 × 10^−4^]; [1 × 10^−4^, 1 × 10^−5^]; [1 × 10^−3^, 1 × 10^−4^, 1 × 10^−5^]. The polygenicity of the region equals the sum of the mixture proportions and the number of causal SNPs are spread equally amongst all the mixture components. For causal effect sizes drawn from the distribution with larger variances (e.g. 1 × 10^−3^), our estimates are relatively unbiased. However, for distributions with smaller variance components (e.g. 1 × 10^−5^), we start to see a downward bias proportional to the fraction of SNPs drawn from the distribution with the smaller variance component(s). (TIF) [file pcbi.1009483.s002.tif]

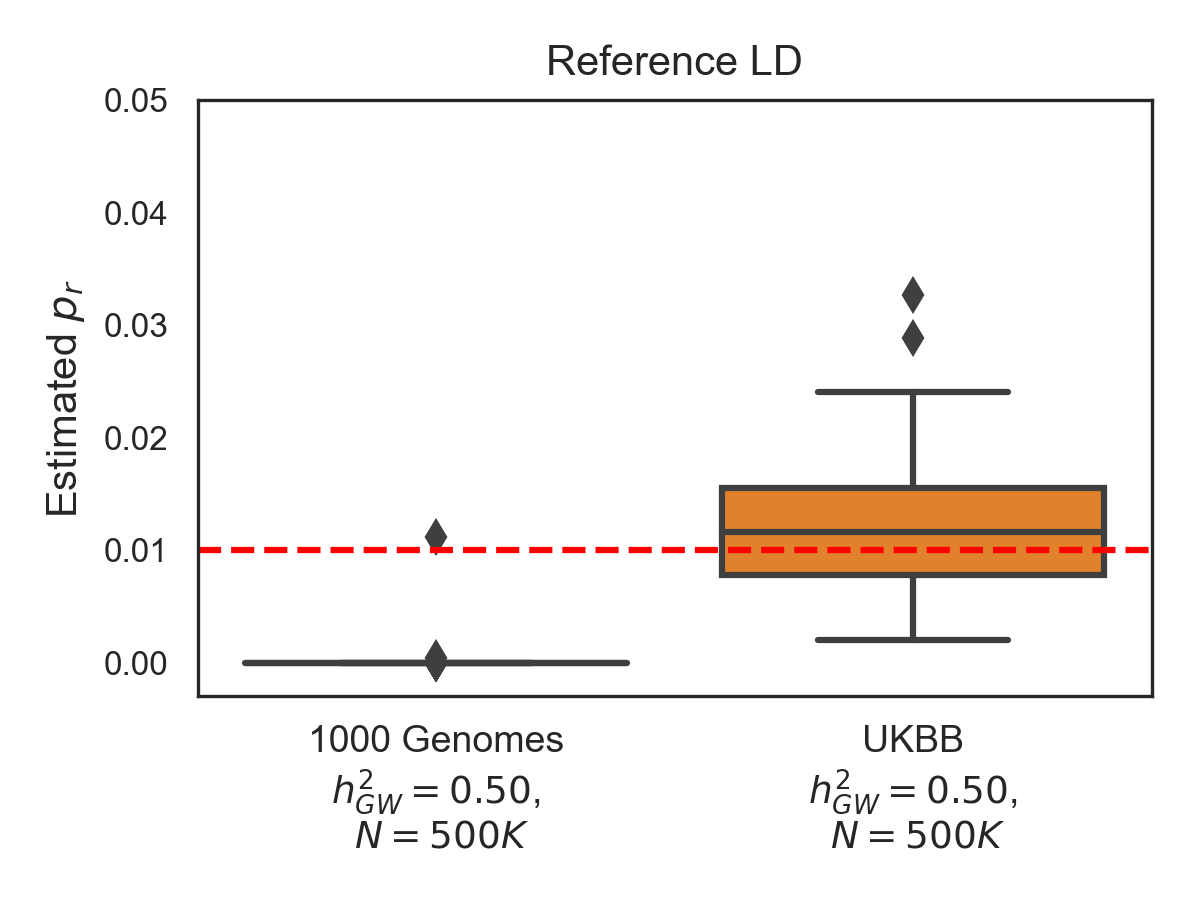

Supplement: S3 Fig — The first GWAS (left) is simulated LD computed with genotypes from the UK Biobank (N = 337, 205) and inference is performed using LD computed from the European individuals from the 1000 Genomes reference panel (N = 503). The second GWAS (right) is simulated with LD derived from a subset of (N1 = 168, 602) genotypes from the UK Biobank and inference is performed using LD computed from a separate, non-overlapping subset (N2 = 168, 602) of individuals also from the UK Biobank. We find that when using LD from separate studies (1000 Genomes), BEAVR fails to accurately estimate the regional polygenicity. However, when we use LD computed from a separate set of individuals from the same study, we find our estimates are approximately unbiased. (TIF) [file pcbi.1009483.s003.tif]

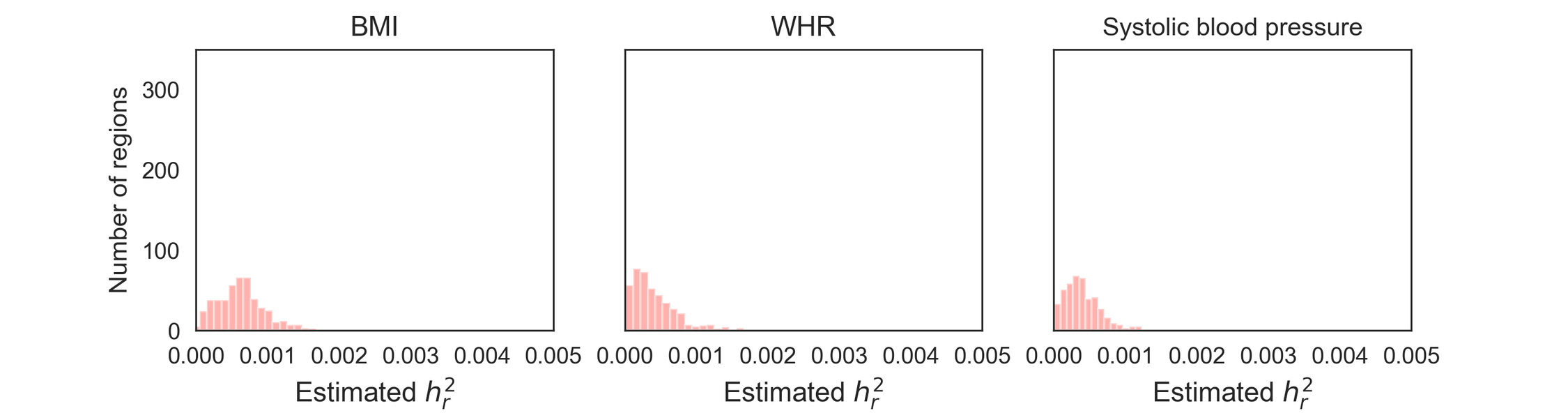

Supplement: S4 Fig — We divide the genome into 6-Mb regions and report the posterior mean of the regional polygenicity for each region across BMI, waist-hip ratio (WHR), and systolic blood pressure. (TIF) [file pcbi.1009483.s004.tif]

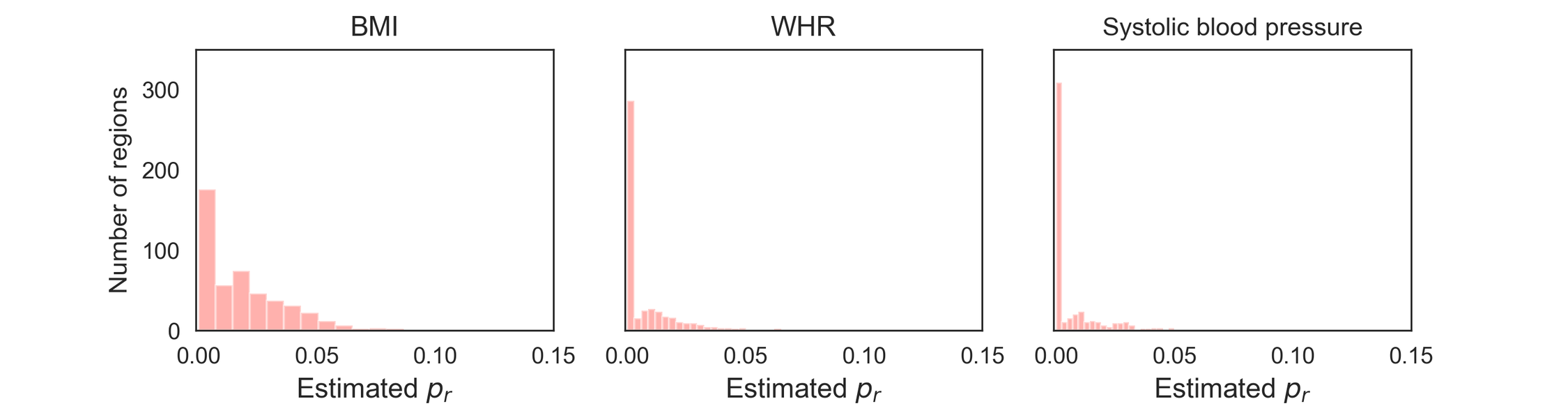

Supplement: S5 Fig — Using external software [12], we estimate the heritability in each 6-Mb region for each trait. We report the distribution of regional heritability for BMI, waist-hip ratio (WHR), and systolic blood pressure. (TIF) [file pcbi.1009483.s005.tif]

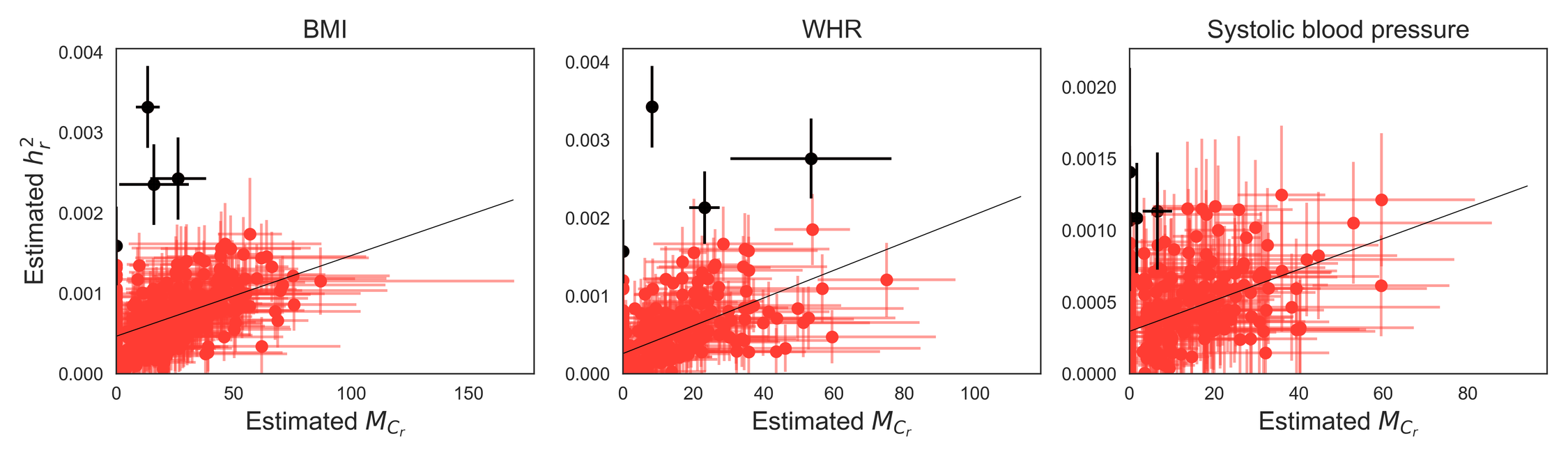

Supplement: S6 Fig — We show the relationship between the number of causal SNPs and heritability for each region across BMI, waist-hip ratio (WHR), and systolic blood pressure. We fit a linear regression for each trait. Horizontal error bars represent two posterior standard deviations around our estimates for the number of causal SNPs. Vertical error bars represent twice the standard error around the estimates of regional heritability. Dots in black denote outlier regions which have an absolute studentized residual larger than 3. (TIF) [file pcbi.1009483.s006.tif]
