# Supplementary material for: Estimation of regional polygenicity from GWAS provides insights into the genetic architecture of complex traits
Source: PLoS Comput Biol. 2021 Oct 21;17(10):e1009483. doi: 10.1371/journal.pcbi.1009483 (PMC8562817; doi:10.1371/journal.pcbi.1009483)
Supplement: S1 File — Additional derivations for the Gibbs sampler. Table A. Linear relationship between the number of causal SNPs and heritability. We model the linear relationship between the number of causal SNPs for a trait and the heritability across all regions of the genome. We report the slope of the regression and the standard error. The slope can be interpreted as the expected per-SNP heritability contribution per causal SNP. The last column reports the number of ‘outlier’ regions for each trait, defined as a region with an absolute studentized residual greater than 3. Table B. Covariates that are associated with regional heritability hr2. We perform a multivariate regression of heritability on the number of SNPs, number of causal SNPs, number of genes, median B-statistic, and non-cell-type-specific annotations [28]. Only the number of causal SNPs (MCr) remains significant for all traits after the multiple testing correction (average p-value = 6.37 × 10^−11^), and the number of SNPs (Mr) remains significant for 3 our of 5 traits after the multiple testing correction. Table C. Likelihood ratio test assessing the role of gene density in regional polygenicity estimates. We perform a likelihood ratio test between the following two models to assess the effect of gene density on the number of causal SNPs (MCr) after adjusting for both regional heritability and the number of SNPs (H0:MCr∼hr2+Mr;H1:MCr∼hr2+Mr+#genes). (PDF) [file pcbi.1009483.s007.pdf]

Supplementary Materials

Additional derivations

Sampling $\gamma_{r,m}, c_{r,m}$

We derive a Gibbs sampler to sample from the posterior distribution of each parameter $\{c_{r,m}, \gamma_{r,m}, p_r\}$. Because the causal status and effect size of a SNP are highly correlated, we sample $(\gamma_{r,m}, c_{r,m})$ together in a block.

Let $\boldsymbol{\theta}_r = \{(\boldsymbol{\gamma}_{\neg r,m}, \boldsymbol{c}_{\neg r,m}), h_r^2, p_r, \alpha\}$, where $\boldsymbol{\gamma}_{\neg r,m}$ denotes all effect sizes except for the effect of the mth SNP; this similarly follows for $\boldsymbol{c}_{\neg r,m}$. We denote $\sigma_{r,g}^2 = \frac{h_r^2}{M_r p_r}$ and $\sigma_e^2 = \frac{1-h_r^2}{N}$. The derivation for each marginal posterior distribution, $P(\gamma_{r,m} \mid \cdot)$ and $P(c_{r,m} \mid \cdot)$ is given separately below. By the chain rule note that:

$$P(\gamma_{r,m}, c_{r,m} \mid \boldsymbol{\theta}_r, \tilde{\boldsymbol{\beta}}_r) = P(\gamma_{r,m} \mid c_{r,m}, \boldsymbol{\theta}_r, \tilde{\boldsymbol{\beta}}_r) P(c_{r,m} \mid \boldsymbol{\theta}_r, \tilde{\boldsymbol{\beta}}_r)$$

Additionally, for convenience we denote $\boldsymbol{r}_{r,m} = \tilde{\boldsymbol{\beta}}_r - \boldsymbol{V}_r^{\frac{1}{2}} \boldsymbol{\gamma}_r \circ \boldsymbol{c}_r + \boldsymbol{V}_{r,m}^{\frac{1}{2}} \gamma_{r,m} c_{r,m}$, which is the residual from subtracting the effects of all SNPs except for SNP m.

Deriving the first term, $P(\gamma_{r,m} \mid c_{r,m}, \boldsymbol{\theta}_r, \tilde{\boldsymbol{\beta}}_r)$, we break up the expression into the cases when $c_{r,m} = 1$ and $c_{r,m} = 0$:

$$
\begin{aligned}
P(\gamma_{r,m} \mid c_{r,m} = 1, \boldsymbol{\theta}_r, \tilde{\boldsymbol{\beta}}_r) \propto & P(\tilde{\boldsymbol{\beta}}_r \mid \gamma_{r,m}, c_{r,m} = 1, \boldsymbol{\theta}_r) P(\gamma_{r,m} \mid \boldsymbol{\theta}_r, c_{r,m} = 1) \\
&= \exp\Big\{ - \frac{1}{2\sigma_e^2} (\boldsymbol{r}_{r,m} - \boldsymbol{V}_{r,m}^{\frac{1}{2}} \gamma_{r,m})^\top (\boldsymbol{r}_{r,m} - \boldsymbol{V}_{r,m}^{\frac{1}{2}} \gamma_{r,m}) \Big\} \exp\Big\{ - \frac{1}{2\sigma_{r,g}^2} \gamma_{r,m}^2 \Big\} \\
&= \exp\left\{ - \frac{1}{2\sigma_e^2} (\boldsymbol{r}_{r,m}^\top \boldsymbol{r}_{r,m} - 2\boldsymbol{r}_{r,m}^T \boldsymbol{V}_{r,m}^{\frac{1}{2}} \gamma_{r,m} + \boldsymbol{V}_{r,m}^{\frac{1}{2}^\top} \boldsymbol{V}_{r,m}^{\frac{1}{2}} \gamma_{r,m}^2) - \frac{1}{2\sigma_{r,g}^2} \gamma_{r,m}^2 \right\} \\
& [\text{drop constants that don't depend on } \gamma_{r,m}] \\
&= \exp\left\{ - \frac{1}{2\sigma_e^2} (-2\boldsymbol{r}_{r,m}^\top \boldsymbol{V}_{r,m}^{\frac{1}{2}} \gamma_{r,m} + \boldsymbol{V}_{r,m}^{\frac{1}{2}^\top} \boldsymbol{V}_{r,m}^{\frac{1}{2}} \gamma_{r,m}^2) - \frac{1}{2\sigma_{r,g}^2} \gamma_{r,m}^2 \right\} \\
& [\text{common denominators}] \\
&= \exp\left\{ - \frac{1}{2\sigma_e^2 2\sigma_{r,g}^2} (-2\boldsymbol{r}_{r,m}^\top \boldsymbol{V_m}^{\frac{1}{2}} 2\sigma_{r,g}^2 \gamma_{r,m} + \boldsymbol{V}_{r,m}^{\frac{1}{2}^\top} \boldsymbol{V}_{r,m}^{\frac{1}{2}} 2\sigma_{r,g}^2 \gamma_{r,m}^2 + 2\sigma_e^2 \gamma_{r,m}^2) \right\} \\
&= \exp\left\{ - \frac{\gamma_{r,m}^2}{2} \left(\frac{1}{\sigma_{r,g}^2} + \frac{1}{\sigma_e^2} \boldsymbol{V}_{r,m}^{\frac{1}{2}^\top} \boldsymbol{V}_{r,m}^{\frac{1}{2}} \right) + \gamma_{r,m} \left(\frac{1}{\sigma_e^2} \boldsymbol{r}_{r,m}^\top \boldsymbol{V}_{r,m}^{\frac{1}{2}} \right) \right\} \\
&= \exp\left\{ - \frac{\gamma_{r,m}^2}{2} (a) + \gamma_{r,m} (b) \right\} \\
& \left(a = -\frac{1}{2\sigma_{r,m}^2}, b = \frac{\mu_{r,m}}{\sigma_{r,m}^2} \right) \\
&= \mathcal{N}(\mu_{r,m}, \sigma_{r,m}^2) \\
& \frac{1}{\sigma_{r,m}^2} = \frac{1}{\sigma_{r,g}^2} + \frac{1}{\sigma_e^2} \boldsymbol{V}_{r,m}^{\frac{1}{2}^\top} \boldsymbol{V}_{r,m}^{\frac{1}{2}} \\
& \mu_{r,m} = \sigma_{r,m}^2 \frac{1}{\sigma_e^2} \boldsymbol{r}_{r,m}^T \boldsymbol{V}_{r,m}^{\frac{1}{2}}
\end{aligned}
$$

$$
P(\gamma_{r,m} \mid c_{r,m} = 0, \boldsymbol{\theta}_r, \tilde{\boldsymbol{\beta}}_r) = \delta_0(\gamma_{r,m})
$$

The bottom line follows because the effect size of a non-causal SNP is 0 ($c_{r,m} = 0$).

Deriving the second term, $P(c_{r,m} \mid \boldsymbol{\theta}_r, \tilde{\boldsymbol{\beta}}_r)$:

$$
\begin{aligned}
P(c_{r,m} = 1 \mid \boldsymbol{\theta}_r, \tilde{\boldsymbol{\beta}}_r) &= \int P(c_{r,m} = 1, \gamma_{r,m} \mid \boldsymbol{\theta}_r, \tilde{\boldsymbol{\beta}}_r) d\gamma_{r,m} \\
&= \int \frac{P(\tilde{\boldsymbol{\beta}}_r \mid \gamma_{r,m}, c_{r,m} = 1, \boldsymbol{\theta}_r) P(\gamma_{r,m}, c_{r,m} = 1 \mid \boldsymbol{\theta}_r)}{P(\tilde{\boldsymbol{\beta}}_r \mid \boldsymbol{\theta}_r)} d\gamma_{r,m} \\
&= \int \frac{P(\tilde{\boldsymbol{\beta}}_r \mid \gamma_{r,m}, c_{r,m} = 1, \boldsymbol{\theta}_r) P(\gamma_{r,m} \mid c_{r,m} = 1 \boldsymbol{\theta}_r) P(c_{r,m} = 1 \mid \boldsymbol{\theta}_r)}{P(\tilde{\boldsymbol{\beta}}_r \mid \boldsymbol{\theta}_r)} d\gamma_{r,m} \\
&= \frac{P(c_{r,m} = 1 \mid \boldsymbol{\theta}_r)}{P(\tilde{\boldsymbol{\beta}}_r \mid \boldsymbol{\theta}_r)} \int P(\tilde{\boldsymbol{\beta}}_r \mid \gamma_{r,m}, c_{r,m} = 1, \boldsymbol{\theta}_r) P(\gamma_{r,m} \mid c_{r,m} = 1, \boldsymbol{\theta}_r) d\gamma_{r,m} \\
&\text{[denominator does not depend on } c_{r,m}\text{]} \\
&= P(c_{r,m} = 1 \mid \boldsymbol{\theta}_r) \int \Big[\frac{1}{\sqrt{2\pi\sigma_{r,m}^2}} \exp\Big\{ -\frac{1}{2\sigma_{r,m}^2} (\gamma_{r,m} - \mu_{r,m})^2 \Big\} \Big] d\gamma_{r,m} \\
&\quad \times \sqrt{2\pi\sigma_{r,m}^2} \exp\Big\{ -\frac{1}{2\sigma_e^2} \boldsymbol{r}_{r,m}^\top \boldsymbol{r}_{r,m} + \frac{1}{2\sigma_{r,m}^2} \mu_{r,m}^2 \Big\} \\
&= P(c_{r,m} \mid \boldsymbol{\theta}_r) \frac{\sqrt{2\pi\sigma_{r,m}^2}}{\sqrt{2\pi\sigma_e^2} \sqrt{2\pi\sigma_{r,g}^2}} \exp\Big\{ -\frac{1}{2} \frac{\boldsymbol{r}_{r,m}^\top \boldsymbol{r}_{r,m}}{\sigma_e^2} + \frac{1}{2\sigma_{r,m}^2} \mu_{r,m}^2 \Big\}
\end{aligned}
$$

To sample $c_{r,m}$, we draw $c_{r,m} \sim \text{Bern}(d_{r,m})$, where $d_{r,m}$ is defined as follows:

$$d_{r,m} = \frac{\frac{\sqrt{2\pi\sigma^2_{r,m}}}{\sqrt{2\pi\sigma^2_e}\sqrt{2\pi\sigma^2_{r,g}}} P(c_{r,m} = 1) \exp\Big\{ -\frac{1}{2}\frac{\boldsymbol{r}^\top_{r,m}\boldsymbol{r}_{r,m}}{\sigma^2_e} + \frac{1}{2\sigma^2_{r,m}}\mu^2_{r,m} \Big\}}{\frac{\sqrt{2\pi\sigma^2_{r,m}}}{\sqrt{2\pi\sigma^2_e}\sqrt{2\pi\sigma^2_{r,g}}} P(c_{r,m} = 1 \mid \boldsymbol{\theta}_r) \exp\Big\{ -\frac{1}{2}\frac{\boldsymbol{r}^\top_{r,m}\boldsymbol{r}_{r,m}}{\sigma^2_e} + \frac{1}{2\sigma^2_{r,m}}\mu^2_{r,m} \Big\} + \frac{1}{\sqrt{2\pi\sigma^2_e}} P(c_{r,m} = 0) \exp\Big\{ -\frac{1}{2\sigma^2_e}\boldsymbol{r}^\top_{r,m}\boldsymbol{r}_{r,m} \Big\}}$$

$$= \frac{\frac{\sqrt{2\pi\sigma^2_{r,m}}}{\sqrt{2\pi\sigma^2_e}\sqrt{2\pi\sigma^2_{r,g}}} (p_r) \exp\Big\{ -\frac{1}{2}\frac{\boldsymbol{r}^\top_{r,m}\boldsymbol{r}_{r,m}}{\sigma^2_e} + \frac{1}{2\sigma^2_{r,m}}\mu^2_{r,m} \Big\}}{\frac{\sqrt{2\pi\sigma^2_{r,m}}}{\sqrt{2\pi\sigma^2_e}\sqrt{2\pi\sigma^2_{r,g}}} (p_r) \exp\Big\{ -\frac{1}{2}\frac{\boldsymbol{r}^\top_{r,m}\boldsymbol{r}_{r,m}}{\sigma^2_e} + \frac{1}{2\sigma^2_{r,m}}\mu^2_{r,m} \Big\} + \frac{1}{\sqrt{2\pi\sigma^2_e}} (1 - p_r) \exp\Big\{ -\frac{1}{2\sigma^2_e}\boldsymbol{r}^\top_{r,m}\boldsymbol{r}_{r,m} \Big\}}$$

[break up terms over exp]

$$= \frac{\frac{\sqrt{2\pi\sigma^2_{r,m}}}{\sqrt{2\pi\sigma^2_e}\sqrt{2\pi\sigma^2_{r,g}}} (p_r) \exp\Big\{ -\frac{1}{2}\frac{\boldsymbol{r}^\top_{r,m}\boldsymbol{r}_{r,m}}{\sigma^2_e} \Big\} \exp\{\frac{1}{2\sigma^2_{r,m}}\mu^2_{r,m} \Big\}}{\frac{\sqrt{2\pi\sigma^2_{r,m}}}{\sqrt{2\pi\sigma^2_e}\sqrt{2\pi\sigma^2_{r,g}}} (p_r) \exp\Big\{ -\frac{1}{2}\frac{\boldsymbol{r}^\top_{r,m}\boldsymbol{r}_{r,m}}{\sigma^2_e} \Big\} \exp\{\frac{1}{2\sigma^2_{r,m}}\mu^2_{r,m} \Big\} + \frac{1}{\sqrt{2\pi\sigma^2_e}} (1 - p_r) \exp\Big\{ -\frac{1}{2\sigma^2_e}\boldsymbol{r}^\top_{r,m}\boldsymbol{r}_{r,m} \Big\}}$$

[common exp terms and constants from top/bottom]

$$= \frac{(p_r)\sqrt{\frac{\sigma^2_{r,m}}{\sigma^2_{r,g}}} \exp\Big\{ \frac{1}{2\sigma^2_{r,m}}\mu^2_{r,m} \Big\}}{(p_r)\sqrt{\frac{\sigma^2_{r,m}}{\sigma^2_{r,g}}} \exp\Big\{ \frac{1}{2\sigma^2_{r,m}}\mu^2_{r,m} \Big\} + (1 - p_r)}$$

In summary, to jointly sample $(\gamma_{r,m}, c_{r,m})$, one first samples $c_{r,m}$. Then depending on if $c_{r,m} = 1$ we sample $\gamma_{r,m}$, and if $c_{r,m} = 0$ we set γ_m to a point mass at 0:

$$c_{r,m} \sim \text{Bern}(d_{r,m})$$

$$\gamma_{r,m} \sim \begin{cases} \mathcal{N}(\mu_{r,m}, \sigma^2_{r,m}) & \text{if } c_{r,m} = 1 \\ 0 & \text{if } c_{r,m} = 0 \end{cases}$$

Trait	OLS slope (CI)	Number outlier regions
BMI	1.078×10^{-5} (1.1×10^{-6})	4
Height	2.874×10^{-5} (1.48×10^{-6})	9
Waist-hip ratio	1.781×10^{-5} (1.27×10^{-6})	4
Diastolic blood pressure	1.337×10^{-5} (1.06×10^{-6})	5
Systolic blood pressure	1.078×10^{-5} (1.06×10^{-6})	5

Table A: Linear relationship between the number of causal SNPs and heritability. We model the linear relationship between the number of causal SNPs for a trait and the heritability across all regions of the genome. We report the slope of the regression and the standard error. The slope can be interpreted as the expected per-SNP heritability contribution per causal SNP. The last column reports the number of 'outlier' regions for each trait, defined as a region with an absolute studentized residual greater than 3.

Trait	Annotation	p-value
BMI	M_{C_r}	1.11E-13
	M_r	3.33E-04
Height	M_{C_r}	1.79E-28
	M_r	4.91E-04
Waist-hip ratio	M_{C_r}	3.73E-21
Diastolic blood pressure	M_{C_r}	6.79E-19
	M_r	3.38E-04
Systolic blood pressure	M_{C_r}	3.18E-10

Table B: Covariates that are associated with regional heritability $\mathbf{h_r^2}$. We perform a multivariate regression of heritability on the number of SNPs, number of causal SNPs, number of genes, median B-statistic, and non-cell-type-specific annotations. Only the number of causal SNPs (M_{C_r}) remains significant for all traits after the multiple testing correction (average p-value $=6.37 \times 10^{-11}$), and the number of SNPs (M_r) remains significant for 3 our of 5 traits after the multiple testing correction.

Trait	p-value
Systolic blood pressure	0.028
Height	3.93×10^{-7}
Waist-hip ratio	0.070
BMI	0.073
Diastolic blood pressure	0.096

Table C: Likelihood ratio test assessing the role of gene density in regional polygenicity estimates. We perform a likelihood ratio test between the following two models to assess the effect of gene density on the number of causal SNPs (M_{C_r}) after adjusting for both regional heritability and the number of SNPs ($H_0 : M_{C_r} \sim h_r^2 + M_r; H_1 : M_{C_r} \sim h_r^2 + M_r + \#\text{genes}$).
