# Supplementary material for: Estimation of regional polygenicity from GWAS provides insights into the genetic architecture of complex traits
Source: PLoS Comput Biol. 2021 Oct 21;17(10):e1009483. doi: 10.1371/journal.pcbi.1009483 (PMC8562817; doi:10.1371/journal.pcbi.1009483)
Supplement: Attachment — Submitted filename: responses.pdf [file pcbi.1009483.s008.pdf]

Comments to the Authors:

Reviewer #1: Major:

1) Figure 2 results I find troublesome. I presume that when p_r has downward bias, so will the estimation of heritability. And if the posterior inclusion probabilities were used as evidence for association, BEAVR will have low power for typical GWAS datasets whose sample sizes are far less than 50k. Although one may argue that the main goal of BEAVR is to estimate polygenicity, my opinion is that effect sizes, heritability, and polygenicity are intimately connected, and it would be different for me to believe it does one better while others worse. Also, why does BEAVR do well with extremely large samples? My guess is that the posterior BEAVR sampled is narrowly picked, so that even a naively implemented sampling scheme can get it right with a relatively small amount of samples.

We thank the reviewer for raising these concerns. We have broken down our response into the following points:

a.) Downward bias of p_r with sample sizes <50K and lack of bias with large sample sizes

In Figure 2, we demonstrate that BEAVR lacks power for studies with sample sizes <50K individuals. BEAVR was designed for biobank-scale studies in mind, which often includes hundreds of thousands of individuals. In these larger sample sizes (e.g. N=500K), estimates of regional polygenicity are unbiased.

BEAVR performs well when there is a large sample size (N) because the sample size factors into the standard error of the estimated GWAS effect sizes:

$$\hat{\beta} \sim \mathcal{N}\left(\mathbf{V}\beta, \mathbf{V}\sigma_e^2\right)$$

$$\sigma_e^2 = \frac{(1 - h_{GW}^2)}{N}$$

As the variance of the GWAS effect size distribution decreases, it becomes easier to accurately estimate the true causal effect sizes (β). Additionally, the number of SNPs (*M*) modulates the effect of the prior on the posterior distribution. As the number of SNPs increase, the likelihood tends to overwhelm the prior in their relative contributions to the posterior distribution. This is demonstrated in Figure 3b where we see that the variance of our estimates increase as the number of SNPs decrease. Additionally in Figure 3c, we demonstrate that the inference of the polygenicity parameter, *p*, is largely invariant to the choice of prior suggesting that the posterior estimated by BEAVR is largely driven by the data.

b) Posterior inclusion probabilities as evidence of association

While the model underlying BEAVR can estimate the per-SNP posterior inclusion probabilities, we emphasize that regional polygenicity estimates are obtained by integrating over the possible causal status of each SNP (so that BEAVR does not estimate regional polygenicity by first estimating the per-SNP posterior inclusion probabilities). Estimating parameters on the per-variant level and adequate benchmarking would require additional analyses that we chose to not include in the study at this time. If the goal is assessing evidence for association per SNP, we suggest that users use BEAVR to estimate regional polygenicity to prioritize regions of interest and then estimate the posterior inclusion probabilities using fine-mapping methods[1–4].

c) Connection between effect sizes, heritability, and polygenicity

Although heritability and polygenicity are inherently connected, the accurate estimation of one quantity is not always dependent on the precise estimation of the other quantities, i.e. heritability and effect sizes. Specifically, heritability can be estimated without making assumptions about other aspects of genetic architecture such as the polygenicity or the coupling between effect sizes and MAF. Indeed, previous methods for estimating heritability[5–7] have used fixed-effect models that accurately estimate SNP heritability despite not making explicit estimates of polygenicity. Another example of some parameters being easier to estimate is demonstrated in Carbonetto and Stephens 2012[8]. While their estimates of posterior inclusion probabilities are not well-calibrated, estimates of the proportion of causal variants is accurate-- demonstrating again that inherently connected parameters in a model can be accurately estimated despite noisy estimates of other model parameters.

2) The authors claim their approach “yields approximately unbiased estimates of regional polygenicity.” This begs the question how does the ascertainment bias on genetic variants affect the estimation of polygenicity. For example, if SNPs of minor allele frequency of 1% were removed, will the polygenicity estimate be similar to SNP maf cutoff of 5%?

Due to ascertainment, not all variants may be present in a dataset (e.g. genotyped variants only, MAF filtered). We clarify our definition of ‘polygenicity’ as the proportion of all ‘causal variants’ which are variants measured within a given GWAS that either have a nonzero effect on the trait or tag unmeasured variants through LD that also have a nonzero effect. The inferred causal effects are statistical rather than indicative of true biological mechanisms. Thus, the estimates of polygenicity are conditional on ascertainment and are defined with respect to the set of variants in the analyzed GWAS, which is similar to the definition of SNP-heritability estimates which are also specific to each set of variants [5,6,9,10]. For example, in an analysis consisting of only SNPs with MAF >5%, then the polygenicity estimate only describes this set of common SNPs. Extrapolating estimates of polygenicity to other sets of SNPs requires additional assumptions which we do not include in this work.

We added the following clarification within the main text under the *Introduction:*

Introduction: lines 66-72:

In this work, we define 'causal variants' as a set of variants measured that have either a nonzero effect on the trait or tag unmeasured variants through LD that also have a nonzero effect. This particular definition does not imply a causal biological relationship nor formal causation as defined in causal inference. Thus, the estimates of polygenicity are defined with respect to the set of variants in the analyzed GWAS, which is similar to the definition of SNP-heritability estimates which are also specific to each set of variants and cannot be extrapolated to other sets of SNPs.

3) How to obtain regional heritability? Estimated by HESS. You may reestimate the regional heritability after you fit the BEAVR, how does it compare to the one estimated by HESS?

We thank the reviewer for this suggestion. We do use HESS to provide a priori estimates of regional heritability for the BEAVR model, which then provides estimates of regional polygenicity. Currently, the model does not include steps for inferring regional heritability, neither with previous estimates of polygenicity or jointly. The model we present is naturally extendable to inferring regional heritability and would require an additional Metropolis-Hastings step to sample from the posterior distribution of the regional heritability parameter. However, the convergence of the sampler, as well as the accuracy of the parameter estimates, is unknown for this proposed model.

Additionally, using HESS-estimates to estimate regional polygenicity, and then using BEAVR-estimated polygenicity to again estimate heritability using BEAVR, would not be directly comparable to the initial HESS-estimated heritability. This is because the BEAVR-estimated heritability would subsequently be dependent on the HESS estimates and would present a circular relationship between the two parameter estimates, making a statistical comparison between the two estimates biased.

Minor:

1) The word "causal" was used casually by the authors.

We thank the reviewer for bringing this to our attention. The definition of "causal" variant in the context of this work describes a variant with a measurable non-zero effect size or tags unmeasured variants through LD that also have a nonzero effect. Additionally, we do not imply any biologically causal relationship nor formal causation such as used in causal inference. This definition of "causal" has been widely used in statistical genetics literature[1,10–15].

We added the following clarification within the main text:

Introduction: lines 66-69:

In this work, we define 'causal variants' as a set of variants measured that have either a nonzero effect on the trait or tag unmeasured variants through LD that also have a nonzero

effect. This particular definition does not imply a causal biological relationship nor formal causation as defined in causal inference.

2) "Heritability enrichments are largely driven by variation in the number of causal SNPs." Is this consistent with the omnigenic model?

We thank the reviewer for suggesting this hypothesis. The ominigenic model posits that due to the small number of core genes, the majority of heritability is driven by peripheral genes that are seemingly unrelated to specific biological mechanisms. Our findings suggest that causal SNPs are distributed approximately uniformly across the genome where 48.9% of 6Mb regions contain at least 5 causal SNPs. Because "core" genes could be interconnected throughout the entire genome, the effect of a single causal SNP could contribute to the total heritability regardless of its proximity to biologically relevant genes, leading to this uniform spread of causal variants across the genome. Additionally, this interconnectivity would support a highly polygenic architecture, which we also observe across the 5 analyzed traits.

We updated the text to include this discussion:

Discussion: lines 257-264

The observed extreme polygenic architecture of complex traits supports the hypothesis that the majority of disease risk variation is driven by a large proportion of the genome. We find that heritability enrichments are largely driven by the number of causal variants and that most of these variants are spread uniformly across the genome. This finding suggests that a large proportion of genes have at least some, although limited, effect on a trait. These findings are consistent with the recently proposed omnigenic model which suggests that disease risk is driven by a combination of a small number of primary 'core' genes and numerous 'peripheral' genes which are connected to core genes via highly interconnected gene networks.

3) Clarify the definition of region independent, and how to account for the different number of variants in each region.

We thank the reviewer for bringing this to our attention. In this work, the term "region independent" refers to regions where there is no long-range LD between regions, although in reality there will still be small correlations between regions. As described in 'Results: Simulations', we simulated an entire chromosome from real genotypes and synthetic phenotypes to reflect true LD patterns. We then divided the chromosome into 6Mb contiguous regions, reflecting the scenario where the boundaries of the regions may intersect regions of LD. However, we find that the small correlations between regions are negligible when estimating regional polygenicity in simulation (Figure 3a).

We also explicitly consider the number of variants in the variance term of the causal effect sizes:

$$\gamma_m \sim \mathcal{N}\left(0, \frac{h^2}{Mp}\right)$$

The model inherently models the number of SNPs in each region and the effect size for each SNP (γ_m) is estimated at each iteration of the MCMC sampler.

4) Bayesian estimation of variants in a region, shouldn't the acronym be BEVAR instead of BEAVR?

When constructing the acronym, we chose to omit prepositions and indefinite articles, resulting in the current spelling.

5) Figure 3. why in (b) larger M has smaller variation? Is that because large M implies less correlation between a random pair of SNPs?

The credible interval of our estimates of regional polygenicity decreases as the number of SNPs (*M*) increases because the larger regions also have a higher number of causal SNPs (when fixing the polygenicity across regions). Thus, there are more data points to inform the posterior distribution. Additionally, if a region is small, there is a larger impact on the estimated polygenicity when misidentifying causal SNPs due to the small denominator of SNPs in the region. For example, for a region with 10 SNPs and true polygenicity p=0.01, detecting the presence of two causal SNPs would result in an absolute error of 0.01. However, in the same scenario with 1000 SNPs, detecting the presence of an extra causal SNP would only have an absolute error of 0.001.

It is also expected that larger regions may have less overall LD when compared to smaller regions. Because LD decreases with genomic distance, it can be expected that SNPs at the edges of the region will have lower correlation if the region is large. However, because the model explicitly models LD within each region, this phenomenon is largely accounted for.

We add this clarification in the main text as well:

Results: lines 158-163

This trend occurs because larger regions have a higher number of SNPs to inform the posterior distribution, meaning that there will be more certainty in the posterior estimates. Additionally, if a region is small, there is a larger impact on the estimated polygenicity when misidentifying causal SNPs due to the small denominator of SNPs in the region. For example, misidentifying a single causal SNP from a set of 10 SNPs will have a greater impact on the polygenicity estimates compared to a set of 1000 SNPs.

6) Line 178. “We additionally used HESS to estimate regional polygenicity.” Is this a typo? Should polygenicity be heritability?

Yes, this is a typo. We thank the reviewer for bringing this to our attention.

7) Does regional polygenicity have anything to do with gene density in the region? You may compare regions on chr13 with regions on chr19.

We thank the reviewer for proposing this hypothesis. To further assess whether or not the gene density in a region impacts the regional polygenicity (or similarly the number of causal SNPs in a region), we perform a likelihood ratio test between the following two models to assess the effect of gene density on the number of causal SNPs after adjusting for both regional heritability and the number of SNPs. (We note that all regions are 6Mb, so the number of genes per region is equivalent to a measurement of gene density.)

$$H_0 : M_{r,c} \sim h_r^2 + M_r$$

$$H_1 : M_{r,c} \sim h_r^2 + M_r + \#genes$$

As shown in Supplementary Table S3, we find that only the likelihood ratio test for height is significant after adjusting for the number of tested traits (p-value < 0.05/5). This observation could be due to the fact that we included all protein-coding genes for all traits in the analysis, regardless of the specific biological mechanism of each gene. For example, when analyzing BMI, one would expect regions with genes related to lipids or metabolism to harbor more causal variants than genes related to seemingly unrelated biological mechanisms. It has also been shown that height is one of the most polygenic traits with numerous causal variants spread throughout the genome[9]. This potential effect of gene density on the polygenicity of height would support the hypothesis that numerous genes throughout the entire genome, regardless of the specific biological function, contribute to the variance of height. This idea that numerous genes, regardless of functional mechanism, have an effect on a trait is related to the recently proposed 'omnigenic' model[16].

**Trait**	**LRT p-value**
Systolic blood pressure	0.028
Height	3.93e-07
Waist-hip ratio	0.070
BMI	0.073
Diastolic blood pressure	0.096

Supplementary Table S3: Likelihood ratio test assessing the role of gene density in regional polygenicity estimates. We perform a likelihood ratio test between the following two models to assess the effect of gene density on the number of causal SNPs $(M_{r,c})$ after adjusting for both regional heritability and the number of SNPs $(H_0 : M_{r,c} \sim h_r^2 + M_r; H_1 : M_{r,c} \sim h_r^2 + M_r + \#\text{genes})$.

8) Line 376. “We ran BEAVR for 1000 iterations with a burn-in of 250 iterations' '. These numbers appear to be too small. How many proposals / updates in each iteration? How is consistency between independent runs?

We utilize a simple heuristic to initialize all of our parameters in the Gibbs sampler, as outlined in the section “Results - Analysis of UK Biobank phenotypes”. This effective initialization scheme helps the sampler converge quickly and with minimal burn-in. In summary:

- To initialize the causal status of each SNP (c_m), we set c_m=1 if variant *m*'s z-score is >=3.5 (or p-value<=5e-4) since we expect true causal SNPs to have a strong association signal. Though this threshold is arbitrarily chosen, we only implement this heuristic at the initialization step and the sampler performs the formal inference.
- Causal effect sizes (γm) are drawn from the prior distribution (see Equation 3).
- The polygenicity (*p*) is set to the proportion of initialized causal SNPs, as selected in the previous step.

The average acceptance proposal (computed across 100 runs) is 3.8% (sd: 0.86%). Although this acceptance percentage is relatively low, we expect this is due to our efficient initialization scheme. Additionally, across 10 independent runs, we find that the sampler is consistent, where the variance of our estimates between runs is only 2.94e-06,

9) The novel elements in computation need to be presented more clearly and convincingly.

We thank the reviewer for the feedback. We have greatly reorganized the description of our results in ‘Results - Computational efficiency’ in order to provide better clarification of the key contribution of our method:

Leveraging sparsity of the genetic architecture to improve the computational efficiency of the Gibbs sampler: lines 336-363

The key computational bottleneck in the Gibbs sampling scheme involves computing the mean of the posterior distribution of the causal effect size at SNP m (Equation 8). Specifically, the matrix computation associated with the residual term, $\mathbf{r}_{r,m} = \tilde{\beta}_r - \mathbf{V}_r^{\frac{1}{2}}\gamma_r \circ \mathbf{c}_r + \mathbf{V}_{r,m}^{\frac{1}{2}}\gamma_{r,m}c_{r,m}$*, naively scales as* $\mathcal{O}(M_r^2)$ *due to the middle term, which is a matrix of size* $M_r \times M_r$ *multiplied by a vector of size* $M \times 1$*. Because this computation must be performed for every SNP, the overall complexity of the sampler is* $\mathcal{O}(M_r^3)$ *if implemented in this straightforward fashion. Below, we will break down the posterior mean term such that the complexity of computing* $\mathbf{r}_{\mathbf{r},\mathbf{m}}$ *will be* $\mathcal{O}(K_r)$*, where* K_r *is the number of causal SNPs in the region, and the complexity of the sampler will be* $\mathcal{O}(K_rM_r)$*. This is accomplished by two steps: i) breaking the equation into terms that do not need to be updated at every iteration of the sampler, ii) leveraging the*

expected sparsity of the true causal vector and only performing computations over the causal SNPs.

Writing out the posterior mean term and expanding, we get:

$$\begin{aligned}
\mu_{r,m} &= \frac{\sigma_{r,m}^2}{\sigma_e^2}\boldsymbol{r}_{r,m}\boldsymbol{V}_m^{\frac{1}{2}} \\
&= \frac{\sigma_{r,m}^2}{\sigma_e^2}\left[\tilde{\beta}_r - \mathbf{V}_r^{\frac{1}{2}}\gamma_r \circ \mathbf{c}_r + \mathbf{V}_{r,m}^{\frac{1}{2}}\gamma_{r,m}c_{r,m}\right]\boldsymbol{V}_{r,m}^{\frac{1}{2}} \\
&= \frac{\sigma_{r,m}^2}{\sigma_e^2}\left[\tilde{\boldsymbol{\beta}}_r - \sum_{m\neq l}^{M}\boldsymbol{V}_{r,l}^{\frac{1}{2}}\gamma_{r,l}c_{r,l}\right]^\top \boldsymbol{V}_{r,m}^{\frac{1}{2}} \\
&= \frac{\sigma_{r,m}^2}{\sigma_e^2}\left[\tilde{\boldsymbol{\beta}}_{\boldsymbol{r}}^\top\boldsymbol{V}_{r,m}^{\frac{1}{2}} - \sum_{l\neq m}^{M}\boldsymbol{V}_{r,l}^{\frac{1}{2}}\boldsymbol{V}_{r,m}^{\frac{1}{2}}\gamma_{r,l}c_{r,l}\right]
\end{aligned}$$

First, we note that some terms in the expanded equation can be pre-computed as a one-time computation and thus not needed at every iteration of the sampler. The first term, $\tilde{\boldsymbol{\beta}}_{\boldsymbol{r}}^\top\boldsymbol{V}_{r,m}^{\frac{1}{2}}$, is composed of the vector of GWAS effect sizes and a vector of the LD matrix corresponding to the mth SNP, neither of which are updated within the sampler. Second, the term $\boldsymbol{V}_{r,l}^{\frac{1}{2}}\boldsymbol{V}_{r,m}^{\frac{1}{2}}$ can also be pre-computed since it is only the product of two columns within the LD matrix. Aside from the variance terms at the beginning of the equation, which are only scalars, the only term that varies at each iteration of the sampler is $\gamma_{r,l}c_{r,l}$ since both the effect size and causal status need to be re-sampled per iteration. Since this term is wrapped in a summation, the complexity of computing $\mu_{r,m}$ is currently $\mathcal{O}(M_r)$. However, even with this simplification, the overall complexity of the sampler is $\mathcal{O}(M_r^2)$ since this mean term must be computed at every SNP.

To further simplify the computation, we can leverage the observation that most complex traits contain only a small proportion of causal SNPs (K_r) in each region. As the sampler converges to the stationary distribution, we would expect the causal status vector ($\mathbf{c}_r$) to be sparse, where $K_r << M_r$. When this occurs, the summation term will only include a few non-zero terms. By only subtracting the non-zero terms, this term is simply reduced to summing over the number of causal variants and the complexity becomes $\mathcal{O}(K_r)$. Even though this computation must be done at each SNP, the overall complexity of the sampler is only $\mathcal{O}(K_r M_r)$ which is tractable under the assumption of $K_r << M_r$. Specifically, the posterior mean term can be updated as:

$$\mu_{r,m} = \frac{\sigma_{r,m}^2}{\sigma_e^2}\left[\tilde{\boldsymbol{\beta}}_{\boldsymbol{r}}^\top\boldsymbol{V}_{r,m}^{\frac{1}{2}} - \sum_{l\neq m, c_{r,l}=1}^{M}\boldsymbol{V}_{r,l}^{\frac{1}{2}}\boldsymbol{V}_{r,m}^{\frac{1}{2}}\gamma_{r,l}c_{r,l}\right]$$

Reviewer #2: Minor comments

1. Although the authors mention that in-sample LD is used throughout, I believe it is critical to understand what happens when reference panel LD estimates are used. Although GWAS summary statistics are widely available, the corresponding in-sample LD sharing is not a practice that is very common. So, for BEAVR to widely applicable, I believe the authors should investigate or at least address in a basic user-case-scenario the problems one might expect when in-sample LD is not available and LD needs to be estimated from reference panel, and how misspecification affects the inference.

We thank the reviewer for bringing this to our attention. To assess the role of reference panel LD, we construct a simulation where we test the following scenarios:

- GWAS is simulated with genotypes from ‘White, British’ individuals from the UK Biobank (N=337,205) and inference is performed using LD computed from the European individuals from 1000 Genomes reference panel (N=503)
- GWAS is simulated with a subset (N1=168,602) of genotypes from ‘White, British’ individuals from the UK Biobank and inference is performed using a separate non-overlapping subset (N2=168,602) of individuals also from the ‘White, British’ group from the UK Biobank.

The first simulation represents the scenario where only reference panel LD from a completely different study is available and is only matched based on continental ancestry. The second simulation reflects the case where a user might have individual-level data available from the same population cohort as the GWAS study group. For example, a user has obtained summary statistics computed using the group of UK Biobank ‘White, British’ individuals, but then uses LD computed from their own set of UK Biobank genotypes allotted to them through their own application. This scenario closely reflects situations where many groups separately apply for freezes of data from the same study yet share GWAS summary statistics across applications.

We performed simulations where GWAS summary statistics were constructed for 100 regions according to the scenarios listed above. We simulate each region to have regional polygenicity, p=0.01, and regional heritability, h2=0.0001 (corresponding to a genome-wide heritability of 0.50). We find that when using LD from separate studies (1000 Genomes), BEAVR fails to accurately estimate the regional polygenicity. However, when we use LD computed from a separate set of individuals from the same study, we find our estimates are approximately unbiased (Supplementary Figure S6).

Accurately modeling LD plays a vital role in disentangling the correlated effect sizes in a region and distinguishing exactly which SNPs have non-zero effect sizes. Our findings show that this step of the inference is sensitive to mismatching populations between GWAS and reference panel populations. Although both reference panels are representative of continental European ancestry, the ancestry groups within 1000 Genomes are sampled from multiple subcontinental ancestries in Europe (Italy, Spain, etc.), whereas the reference panel constructed from the UK Biobanks is very ancestrally homogeneous in comparison. Our findings show that one can

perform inference using a reference panel constructed from a separate set of individuals than used in the GWAS when both sets of individuals are from the same study (e.g. UK Biobank). These findings suggest that LD reference panels cannot solely be matched on the continental ancestry level but need to be matched on a much finer scale. Additionally, differences in study design between LD reference panel and GWAS studies may also contribute to discrepancies between LD structure.

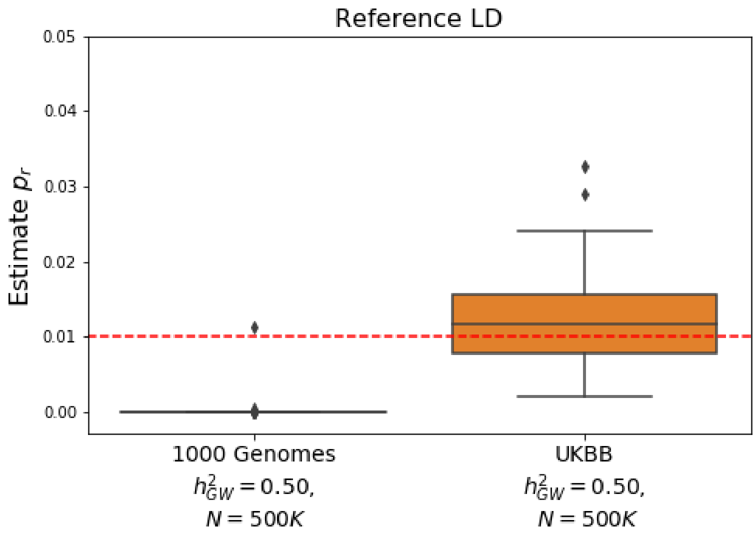

Supplementary Figure S6: Assessing the role of reference LD in estimating regional polygenicity. The first GWAS (left) is simulated with genotypes from the UK Biobank (N=337,205) and inference is performed using LD computed from the European individuals from 1000 Genomes reference panel (N=503). The second GWAS (right) is simulated with LD computed from a subset (N1=168,602) of genotypes from the UK Biobank and inference is performed using LD estimated from a separate non-overlapping subset (N2=168,602) of individuals also from the UK Biobank. We find that when using LD from separate studies (1000 Genomes), BEAVR fails to accurately estimate the regional polygenicity. However, when using LD computed from a separate set of individuals from the same study, we find our estimates are approximately unbiased.

2. Can the authors provide any intuition as to why the inference does not depend at all on \alpha parameter?

We thank the reviewer for raising this concern. The alpha term is integrated within the inference when performing the Metropolis-Hastings step to estimate the polygenicity parameter *p.* Computing the likelihood of *p* includes all SNPs, overwhelming the prior.

3. For highly unbalanced case-control ratio disease phenotypes, the effect sizes/scores might not follow a Gaussian distribution. Since normality is an assumption, can the authors state as to how deviations from normality distort the estimates? Is the method still valid?

We thank the reviewer for raising this concern. To assess the effect of a non-Gaussian causal effect size distribution, we simulate effect sizes from various mixtures of Gaussian distributions (which have heavier tails than a Gaussian distribution). We test a variety of true causal effect size distributions as follows, where the polygenicity of the region equals the sum of the mixture proportions.

- $p_1 N(0, 1 \times 10^{-3}) + p_2 N(0, 1 \times 10^{-4})$
- $p_1 N(0, 1 \times 10^{-4}) + p_2 N(0, 1 \times 10^{-5})$
- $p_1 N(0, 1 \times 10^{-3}) + p_2 N(0, 1 \times 10^{-4}) + p_3 N(0, 1 \times 10^{-5})$

In Supplementary Figure S5, we show the distribution of our estimates of regional polygenicity across the 3 causal effect size distributions where we assume a sample size of N=500K, total regional polygenicity p=0.01, and assume the true heritability of the region is known. The causal SNPs are spread equally amongst all the mixture components. We see that for causal effect sizes drawn from the distribution with larger variances (e.g. 1e-3), our estimates are relatively unbiased. However, for distributions with smaller variance components (e.g. 1e-5), we start to see a downward bias proportional to the fraction of SNPs drawn from the distribution with the smaller variance component(s). Thus, it is not necessarily the exact shape of the distribution of effect sizes, but the magnitude of the causal effect sizes, which affects the accuracy of the estimates. Previous simulations, as shown in Figure 2, demonstrate how the relationship between regional polygenicity and heritability effectively influence the magnitude of the causal effect sizes.

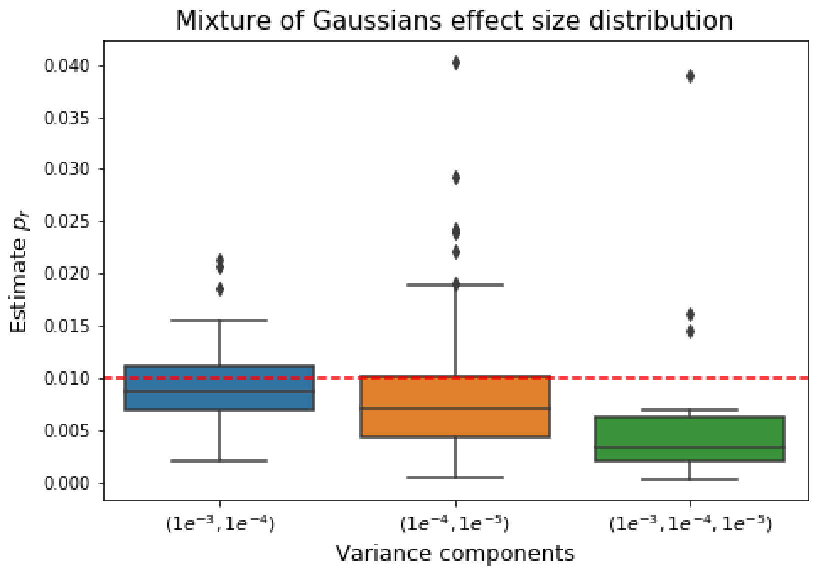

Supplementary Figure S5: Inferring regional polygenicity from non-Gaussian causal effect size distributions. We simulate effect sizes from mixture of Gaussian distributions with the following set of variance components: [1e-3, 1e-4]; [1e-4, 1e-5]; [1e-3, 1e-4, 1e-5]. The polygenicity of the region equals the sum of the mixture proportions and the number of causal SNPs are spread equally amongst all the mixture components. For causal effect sizes drawn from the distribution with larger variances (e.g. 1e-3), our estimates are relatively unbiased. However, for distributions with smaller variance components (e.g. 1e-5), we start to see a downward bias proportional to the fraction of SNPs drawn from the distribution with the smaller variance component(s).

Bibliography

1. Hormozdiari, F., Kostem, E., Kang, E. Y., Pasaniuc, B. & Eskin, E. Identifying causal variants at loci with multiple signals of association. *Genetics* **198**, 497–508 (2014).
2. Kichaev, G. *et al.* Integrating functional data to prioritize causal variants in statistical fine-mapping studies. *PLoS Genet.* **10**, e1004722 (2014).
3. Benner, C. *et al.* FINEMAP: efficient variable selection using summary data from genome-wide association studies. *Bioinformatics* **32**, 1493–1501 (2016).
4. Schaid, D. J., Chen, W. & Larson, N. B. From genome-wide associations to candidate causal variants by statistical fine-mapping. *Nat. Rev. Genet.* **19**, 491–504 (2018).
5. Shi, H., Kichaev, G. & Pasaniuc, B. Contrasting the Genetic Architecture of 30 Complex Traits from Summary Association Data. *Am. J. Hum. Genet.* **99**, 139–153 (2016).
6. Hou, K. *et al.* Accurate estimation of SNP-heritability from biobank-scale data irrespective of genetic architecture. *Nat. Genet.* **51**, 1244–1251 (2019).
7. Wu, Y. & Sankararaman, S. A scalable estimator of SNP heritability for biobank-scale data. *Bioinformatics* **34**, i187–i194 (2018).
8. Carbonetto, P. & Stephens, M. Scalable Variational Inference for Bayesian Variable Selection in Regression, and Its Accuracy in Genetic Association Studies. *Bayesian Analysis* vol. 7 (2012).
9. Yang, J. *et al.* Common SNPs explain a large proportion of the heritability for human height. *Nat. Genet.* **42**, 565–569 (2010).
10. Bulik-Sullivan, B. K. *et al.* LD Score regression distinguishes confounding from polygenicity in genome-wide association studies. *Nat. Genet.* **47**, 291–295 (2015).
11. Holland, D. *et al.* Beyond SNP heritability: Polygenicity and discoverability of phenotypes estimated with a univariate Gaussian mixture model. *PLoS Genet.* **16**, e1008612 (2020).
12. Moser, G. *et al.* Simultaneous discovery, estimation and prediction analysis of complex

traits using a bayesian mixture model. *PLoS Genet.* **11**, e1004969 (2015).

13. O'Connor, L. J. The distribution of common-variant effect sizes. *Nat. Genet.* **53**, 1243–1249 (2021).
14. Shi, H. *et al.* Localizing Components of Shared Transethnic Genetic Architecture of Complex Traits from GWAS Summary Data. *Am. J. Hum. Genet.* **106**, 805–817 (2020).
15. Extreme Polygenicity of Complex Traits Is Explained by Negative Selection. *Am. J. Hum. Genet.* **105**, 456–476 (2019).
16. Boyle, E. A., Li, Y. I. & Pritchard, J. K. An Expanded View of Complex Traits: From Polygenic to Omnigenic. *Cell* **169**, 1177–1186 (2017).
